# Structure-based virtual screening and biological evaluation of novel small-molecule BTK inhibitors

**DOI:** 10.1080/14756366.2021.1999237

**Published:** 2021-12-11

**Authors:** Tony Eight Lin, Li-Chin Sung, Min-Wu Chao, Min Li, Jia-Huei Zheng, Tzu-Ying Sung, Jui-Hua Hsieh, Chia-Ron Yang, Hsueh-Yun Lee, Er-Chieh Cho, Kai-Cheng Hsu

**Affiliations:** a Graduate Institute of Cancer Biology and Drug Discovery, College of Medical Science and Technology, Taipei Medical University, Taipei, Taiwan; b Master Program in Graduate Institute of Cancer Biology and Drug Discovery, College of Medical Science and Technology, Taipei Medical University, Taipei, Taiwan; c Division of Cardiology, Department of Internal Medicine, Shuang Ho Hospital, Taipei Medical University, New Taipei City, Taiwan; d Division of Cardiology, Department of Internal Medicine, School of Medicine, College of Medicine, Taipei Medical University, Taipei, Taiwan; e School of Pharmacy, College of Medicine, National Taiwan University, Taipei, Taiwan; f School of Pharmacy, College of Pharmacy, Taipei Medical University, Taipei, Taiwan; g Biomedical Translation Research Center, Academia Sinica, Taipei, Taiwan; h Division of the National Toxicology Program, National Institute of Environmental Health Sciences, National Institutes of Health, Durham, NC, USA; i Master Program in Clinical Genomics and Proteomics, College of Pharmacy, Taipei Medical University, Taipei, Taiwan; j Cancer Center, Wan Fang Hospital, Taipei Medical University, Taipei, ROC; k Program in Drug Discovery and Development Industry, College of Pharmacy, Taipei Medical University, Taipei, ROC; l TMU Research Center of Cancer Translational Medicine, Taipei Medical University, Taipei, Taiwan; m TMU Research Center of Drug Discovery, Taipei Medical University, Taipei, Taiwan; n Ph.D. Program for Cancer Molecular Biology and Drug Discovery, College of Medical Science and Technology, Taipei Medical University, Taipei, Taiwan

**Keywords:** Virtual screening, kinase inhibitor, BTK, solid tumours, small-molecule inhibitors

## Abstract

Bruton tyrosine kinase (BTK) is linked to multiple signalling pathways that regulate cellular survival, activation, and proliferation. A covalent BTK inhibitor has shown favourable outcomes for treating B cell malignant leukaemia. However, covalent inhibitors require a high reactive warhead that may contribute to unexpected toxicity, poor selectivity, or reduced effectiveness in solid tumours. Herein, we report the identification of a novel noncovalent BTK inhibitor. The binding interactions (i.e. interactions from known BTK inhibitors) for the BTK binding site were identified and incorporated into a structure-based virtual screening (SBVS). Top-rank compounds were selected and testing revealed a BTK inhibitor with >50% inhibition at 10 µM concentration. Examining analogues revealed further BTK inhibitors. When tested across solid tumour cell lines, one inhibitor showed favourable inhibitory activity, suggesting its potential for targeting BTK malignant tumours. This inhibitor could serve as a basis for developing an effective BTK inhibitor targeting solid cancers.

## Introduction

Protein kinases are enzymes that can facilitate protein phosphorylation. Protein phosphorylation is a critical cellular process and is necessary in maintaining cellular homeostasis. Abnormal activation of protein kinases disrupts the normal cell function that can induce tumorigenic features, such as cell proliferation, migration capacity, survival, and angiogenic capacities of cancer^1^^,^^2^. These characteristics make protein kinases important targets for treating various diseases. One such kinase is Bruton’s tyrosine kinase (BTK)^2^.

BTK is a member of the Tec-family of non-receptor tyrosine kinases and has essential roles in regulating proliferation, migration, survival, and B-cell homing^3^. The dysregulation of BTK, therefore, can lead to various haematologic malignancies^4^^,^^5^. However, recent studies have also implicated aberrant BTK signalling in solid cancers^6^^,^^7^. High expression of BTK in glioma can indicate poor survival^6^. Overexpression of a BTK isoform has been observed in patients with colorectal cancer^8^^,^^9^. BTK is also found to be overexpressed in gastric carcinoma and treatment with a BTK inhibitor can reduce tumour growth^10^. The involvement of BTK in these solid tumours may be due to its ability to modulate multiple signalling pathways^2^. Nevertheless, aberrant BTK expression in various malignancies makes it a promising therapeutic target.

The first-in-class BTK inhibitor approved by the Food and Drug Administration (FDA) in the US was ibrutinib for the treatment of chronic lymphocytic leukaemia (CLL) and mantle cell lymphoma^11^. Other small molecules have followed, such as acalabrutinib and zanubrutinib^12^. The FDA-approved BTK inhibitors are orally administered. However, these inhibitors are used primarily for haematological malignancies^11^. Recent studies have evaluated the anti-cancer activity of BTK inhibitors in solid tumours. Unfortunately, the current BTK inhibitors display limited selectivity^7^^,^^13^. This may be due to its covalent binding mechanism, which may bind unselectively to other cysteine-containing kinases^13^^,^^14^. An inhibitor with noncovalent binding would allow researchers to identify a new scaffold that could potentially avoid the side effects associated with the aforementioned covalent inhibitors or serve as a basis for designing inhibitors with greater selectivity towards BTK.

In this study, we performed a structure-based virtual screening (SBVS) approach to identify a novel BTK inhibitor. We identified essential protein-ligand interactions within the target protein binding site. Analysis of these interactions was applied to the SBVS and to increase the potential virtual screening hit rate^15^. For example, small-molecule kinase inhibitors targeting the ATP binding site typically form hydrogen bonds with hinge residues^16^. Filtering compounds based on both their docking scores and binding interactions has proven a useful strategy to not only better understand the protein-ligand binding mechanism, but to improve the hit rate for potential inhibitors^17^. Herein, we identified binding interactions within the BTK binding site by docking known BTK inhibitors. The screening protocol used in this study showed a favourable enrichment factor (EF), validating the screening protocol used. Around 280,000 compounds from the National Cancer Institute (NCI) database were then docked and virtually screened based on their docking and binding score. The top-ranked compounds were selected for experimental validation with enzymatic assays. This led to the identification of NSC726558. This hit compound reduced cell viability of colorectal cancer cells. To further our understanding of the hit compound, the structural analogues of NSC726558 were also tested in the enzymatic assay and we identified a compound that displayed greater enzymatic potency. Cellular assays showed that the hit compounds can reduce cell viability of various solid cancer cell lines. Together, a novel BTK inhibitor was identified and may serve as a basis for further therapeutic investigation.

## Materials and methods

### Molecular docking and compound library preparation

The molecular docking software LeadIT^18^ was used to screen potential inhibitors. The BTK (PDB ID: 5KUP) structure was obtained from the Protein Data Bank^19^. The BTK binding site was prepared in LeadIT version 2.3.2  (BioSolveIT GmbH, Sankt Augustin, Germany). The binding site was set as 10 Å from the co-crystal ligand. The docking parameters used the default settings. All docking procedures were performed on a Microsoft Windows server with 2 CPUs (Intel Xeon E5-2697V2) and 128gb DDR RAM.

The NCI compound database (∼280,000 compounds) was screened for potential BTK inhibitors. The NCI compounds were filtered if they violated the Lipinski Rule of Five. Compounds were then removed if they exhibited Pan Assay Interference Compounds (PAINS) structures. PAINS structures often display false positives in high-throughput screens^17^. The Lipinski Rule of Five estimates pharmacokinetics of compounds in the human body^20^. PAINS are compounds that typically give false-positive results^21^. Next, compounds were docked into the BTK binding site. The top 1000 compounds were selected based on their docking scores. The compounds were then sorted based on scores of binding interactions (see below). The top 300 compounds were selected for kinase inhibition assay; however, based on availability, 11 compounds were selected for further testing.

### Binding interactions

A set of known BTK inhibitors were obtained from BindingDB^22^ and prepared using Pipeline Pilot^23^. Compounds were filtered if their IC_50_ values were greater than 1 µM and structures were grouped using the dissimilarity method in the “Diverse Molecules” component from Pipeline Pilot. A total of 30 known BTK inhibitors with diverse structures were obtained and docked into the BTK binding site. The docking pose of the 30 known BTK inhibitors was analysed to identify possible binding interactions. This study recognises a binding interaction if a residue was observed to form an interaction with ≥ 50% of the BTK inhibitors^17^. The calculation for binding interactions is as follows:

S(i)=N(i)+(−0.01)D(i)

where the binding interaction score, *S*(*i*), for a compound, *i*, equals the number of binding interactions the compound forms, *N*(*i*), plus the docking score , *D*(*i*), of a compound *i* generated using LeadIT. Potential BTK inhibitors were selected for further testing based on their binding interaction score and compound availability.

### Calculation of the enrichment factor

The EF was calculated as follows:

$$\mathrm{EF}=\frac{\left(a/n\right)}{\left(A/N\right)}$$

where *a* is the number of actives found in a sample size (*n*), and *A* is the number of the total actives found in the total number of compounds (*N*), which includes both the decoys and actives. Decoy compounds consisted of 990 randomly selected compounds from the Available Chemical Directory (ACD)^24^. Active compounds in this dataset contained the 30 known BTK inhibitors used to identify binding interactions above. Compounds were combined and docked to ascertain the EF value.

### Kinase inhibition assay

The biochemical assay, Z’LYTE by ThermoFisher Scientific (http://www.thermofisher.com/kinaseprofiling), was used to test kinase activity of identified compounds. In brief, test compounds were prepared in 1% DMSO (final). The test compounds were combined with a kinase mixture and ATP solution that was diluted to 2X and 4X working concentration, respectively, and incubated for 1 h. The development reagent solution was added and measured on a fluorescence plate reader. The assay was performed in duplicate.

### Cell culture

Human cell lines include colon cancer cells DLD1, HT29, and HCT116, glioblastoma cells U118MG and T98G, breast cancer cells MDAMB231 and MCF7, lung cancer cells H1299 and A549, and non-cancerous kidney cells 293 T, were from ATCC. Cells were cultured in RPMI or in DMEM culture medium^25^^,^^26^. Culture medium was supplemented with 10% FBS with 1% antibiotic, and cells were incubated at 37 °C with 5% CO_2_.

### MTT cell proliferation assay

Cell lines were seeded in 96 well plates for overnight, and then treated with different concentrations of the compounds for indicated time points. Cell viability was then analysed by MTT ((3–(4, 5-Dimethylthiazol-2-yl)-2,5-diphenyltetrazolium-bromide) assay (Goldbio, St. Louis, MO)^27^ with microplate spectrophotometer (BioTek Instruments, Winooski, VT). IC_50_ was analysed and determined by SigmaPlot or Graphpad Prism software (GraphPad Software, La Jolla, CA).

### Live and death assay

Calcein-AM (Cayman, Ann Arbor, MI) and propidium iodide (PI) (Biotium, Fremont, CA) were dissolved in DMSO and then diluted in PBS buffer. Cancer cells were seeded in 96 well plate for overnight, and then treated with indicated compound(s) for 48 h. After the treatment, the medium was removed from each well, and the cells were stained with 3 µM calcein-AM and 3 µM PI for 10–20 min. Finally, cells were examined and pictured under the fluorescence microscope at 490nm^26^.

### Flow cytometry

Cells were seeded in 6 well plates for overnight, and treated with NSC725686 compound for 48 h. After the treatment, cells were harvested and fixed by pre-chilled 70% EtOH and stored at −20 °C for overnight, washed with pre-chilled PBS, stained with PI, and finally analysed by flow cytometer (FACS Calibre, BD, Franklin Lakes, NJ)^28^.

### Western blot assay

Cancer cells were seeded into 10 cm dish for overnight, and treated with indicated compounds at indicated concentrations for 48 h. After the treatments, cells were harvested and proteins were extracted. Protein expression was then analysed by western blot with indicated antibodies. Signals were at the end detected by enhanced chemiluminescence (ECL)^29^. PARP, Caspase 3, LC3B, and GAPDH antibodies were from GeneTex. BTK, P-BTK, JNK, and P-JNK antibodies were from Santa Cruz Biotechnology, SantaCruz, CA. GAPDH was used as loading control.

### Calculation of compound properties

Properties for Absorption, Distribution, Metabolism, Elimination, and Toxicity (ADMET) were calculated using Biovia Pipeline Pilot^30^. Compounds were passed through the ADMET All Models, Molecular Weight, Drug likeness (QED), and Lipinski Filter nodes. All components were run using default parameters.

### Immunofluorescence staining (LC3B staining)

HCT116 cells were seeded into 96 well plate for overnight, treated with 20 µM NSC725686 compound for 48 h, and analysed by Immunofluorescence assay^31^^,^^32^. In brief, after the treatment, cells were washed with PBS, fixed, permeabilised, and blocked by BSA. Then, cells were stained with LC3B antibody (GeneTex, Irvine, CA). Finally, cells were analysed and pictured under the fluorescence microscope.

### Statistical analysis

Representative data were shown in figures, and experiments were repeated in at least triplicate under each condition. Data were analysed by one-way ANOVA or Student t-test. Significant data were marked as “*” when *p* values < .05.

## Results and discussion

### Identification of binding interactions

Studies have linked aberrant BTK expression with various cancer malignancies, such as colorectal cancer or gastric carcinoma^8–10^. In this study, we sought to perform an SBVS approach to identify novel BTK inhibitors. While there has been success with virtual screening, further improvements to the hit rate can be made. Members of the human kinome contain a conserved binding site, which can potentially complicate the screening process^33^. Identifying binding interactions for a target binding site may enhance the virtual screening hit rate^15^. To that end, binding interactions within BTK were identified by docking known inhibitors. A set of known BTK inhibitors exhibiting an IC_50_ value of 1 µM or less were obtained from BindingDB^22^. The compounds were further filtered using the “Diverse Molecules” component in Pipeline Pilot to yield 30 known BTK inhibitors^34^. These diverse structures were then docked into the BTK binding site to analyse their binding interactions.

**Figure 1.**
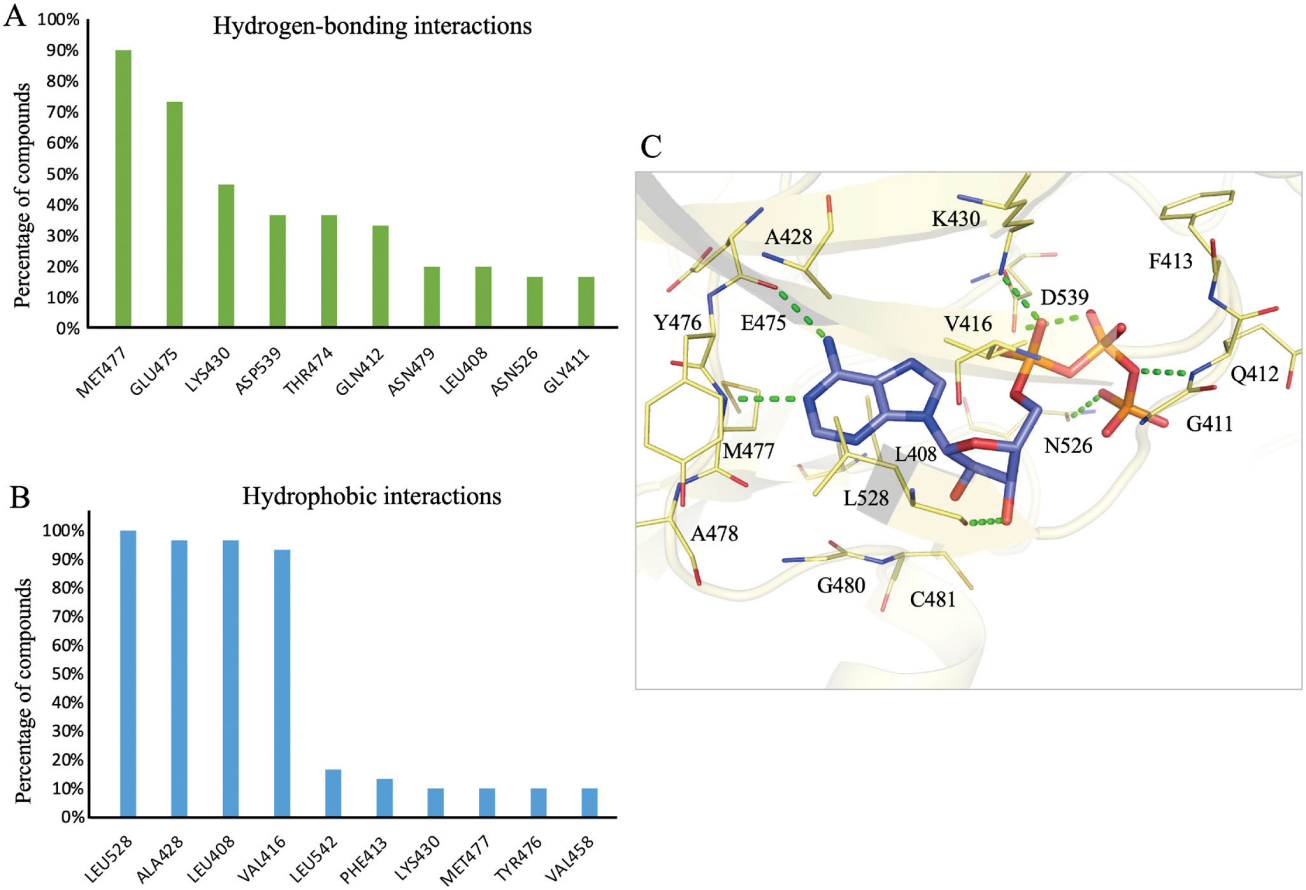
Binding interactions of BTK. (A) hydrogen-bonding and (B) hydrophobic interactions between BTK inhibitors and residues. Residue interactions with ≤ 50% compounds were identified as binding interactions. (C) The binding pose of ATP (purple) in the BTK binding site (yellow). Green dashes denote hydrogen bonds. Residues are labelled and shown as lines.

For our study, a binding interaction was considered if ≥ 50% of the 30 diverse BTK inhibitors formed an interaction with a binding site residue^17^. As a result, two hydrogen-bonding and four hydrophobic interactions were identified (Figure 1(A,B)). Small-molecule inhibitors targeting the conserved kinase binding site commonly form hydrogen bonds with hinge residues^16^. The two hydrogen-bonding interactions consist of hinge residues E475 and M477, forming hydrogen bonds with 90% and 73% of the known BTK inhibitors, respectively (Figure 1(A)). This pattern can be observed upon analysing the docking poses of the inhibitors (Supplementary Figure 1). For example, BTK inhibitors 111589, 111948, and 50094659 form hydrogen bonds with two hinge residues, E475 and M477, while inhibitor 50357330 form a single hydrogen bond to the hinge residue M477. These compounds effectively occupied the BTK binding site. Interestingly, when ATP was docked into the binding site, the adenosine structure formed hydrogen bonds to these hinge residues (Figure 1(C)). The phosphate group of ATP forms hydrogen bonds with residue K430. While residue K430 is the third most common hydrogen bonding residue, it did not meet our ≥50% cut-off (Figure 1(A)).

The known BTK inhibitors formed hydrophobic interactions with four residues with a frequency greater than 90% (Figure 1(B)). These residues form a hydrophobic pocket that sandwich the ATP adenosine structure (Figure 1(C)). For example, residue L528 contains an aliphatic isobutyl side chain that generates hydrophobic interactions with the ATP adenine ring. Residue A428 creates hydrophobic contact with the ring structure. Residues V416 and L408 are located near the ribose ring. Hydrophobic interactions, as well as hydrogen bonds, between one of the ribose hydroxyl groups and L408 occur with this ribose ring of ATP. The heterocyclic rings of BTK inhibitors 111589, 111948, 50094659, and 50357330 are also sandwiched by these hydrophobic residues (Supplementary Figure 1). These hydrophobic binding interactions reveal a preference for a structure that can mimic the adenosine moiety of ATP and can be used with the SBVS filtering criteria to identify potential BTK inhibitors.

An EF analysis was performed to validate the screening protocol used in this study. The EF value will indicate whether the performance of the virtual screening protocol is improved when compared to a random selection^35^. This is a widely used validation technique for assessing the quality of a given virtual screening protocol. The EF value is presented as a ratio of actives in the hit list in a percentage of the tested dataset. A set containing 990 “decoy” compounds from the ACD were combined with the 30 known BTK inhibitors used to identify binding interactions^17^^,^^24^. The EF value at 10% of the active compounds was 11.33 (Supplementary Table 1), suggesting that true positive hits can be identified in the top-ranked compounds using the screening protocol.

### Identification and selection of potential BTK inhibitors

Compounds from the NCI database (roughly 280,000 compounds) were virtually screened for potential BTK inhibitors. The compounds were first filtered based on the Lipinski Rule of Five, which evaluates a compound druglikeness^20^. Compounds containing PAINS substructures, which may lead to false-positive hits, were also removed^21^. Finally, the remaining compounds were docked into the BTK binding site using the molecular docking software LeadIT^18^. Because of the propensity of kinase inhibitors forming hydrogen bonds to the hinge region, docked compounds not displaying these interactions were removed^16^^,^^17^. The remaining compounds were then ranked based on their docking scores, with the top 1000 compounds selected. Compounds were then sorted based on their binding interaction scores. Finally, the top 300 compounds were chosen for further analysis. Based on availability, 11 compounds were selected for enzyme-based assays. Of these, compound NSC726558 showed an inhibition percentage of 59% at a concentration of 10 µM (Supplementary Figure 2). As a result, NSC726558 was selected for further analysis.

### Interaction analysis of selected compound

**Figure 2.**
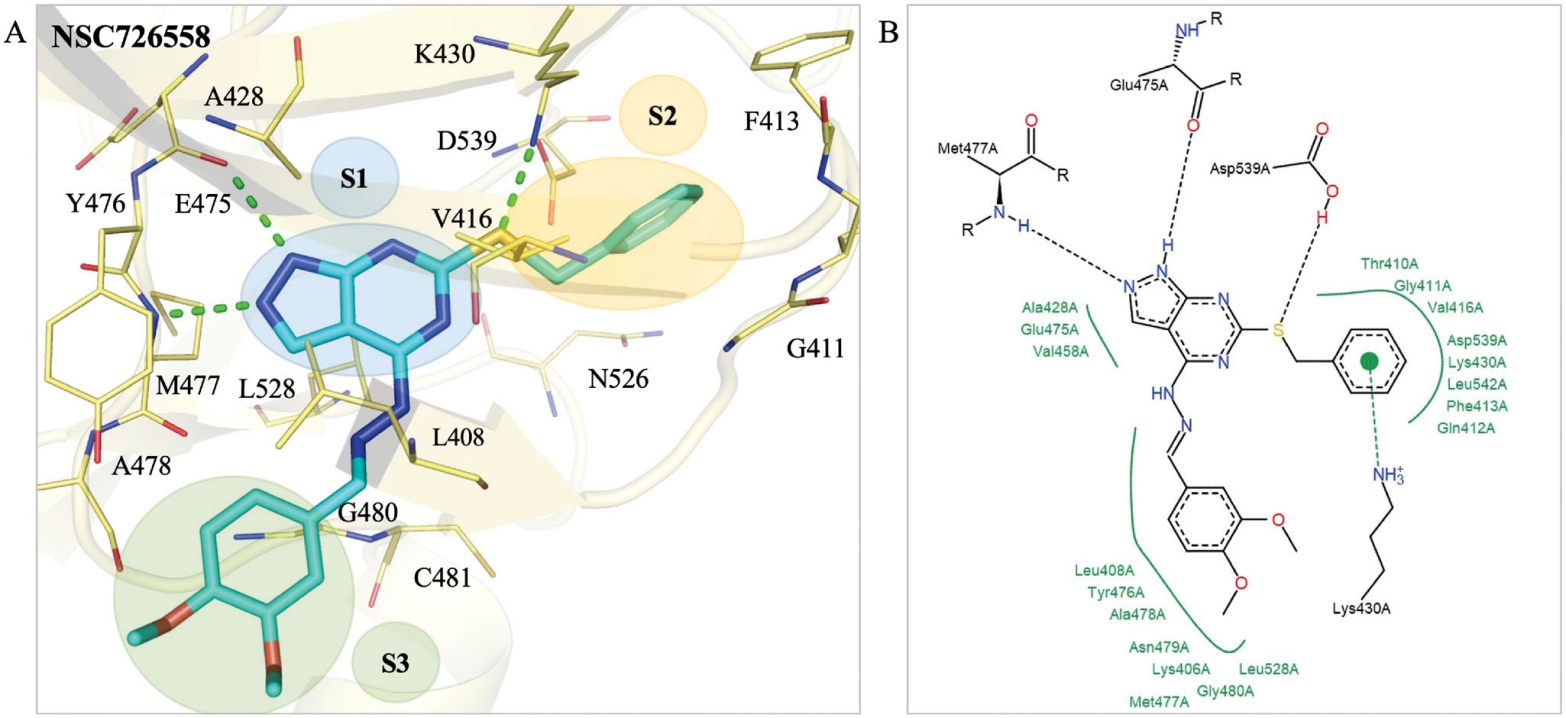
Interaction analysis of compound NSC726558. (A) The docking pose of compound NSC726558 (blue) in the BTK binding site (yellow). Residues are labelled and shown as lines. Green dashes denote hydrogen bonds. (B) The docking pose in 2D of NSC726558 in the BTK binding site. Black dash lines denote hydrogen bonds. Hydrophobic pockets are resented by a green spline and *π* interaction is shown as a green dashed line.

To better understand the binding mechanism, we performed an interaction analysis of compound NSC726558. For clarity, the compound is separated into three distinct sites (S1, S2, and S3) based on their location within the BTK binding site. At S1, a 1H‐pyrazolo[3,4‐d]pyrimidine core occupies the ATP adenosine pocket (Figure 2(A)). As a result, the S1 site is closest to the hinge residue and forms hydrogen bonds to residues E475 and M477. These interactions, which were identified as binding interactions, are facilitated by the nitrogen atoms located on the pyrazolo[3,4‐d]pyrimidine (Figure 2). An additional hydrophobic interaction to residue A428 was also observed within the S1 site. The S2 site is occupied by a phenylmethanethiol moiety that occupies a hydrophobic pocket. This includes a hydrophobic interaction to residue V416. The sulphur atom forms a hydrogen bond with residue D539 (Figure 2). A hydrogen bond was also observed between residue D539 and ATP (Figure 1(C)). Residue D539 forms part of the DFG motif and is pointed inwards towards the binding site^36^. Interactions with residue D539 may contribute to inhibition. However, residue D539 did not meet the threshold as a binding interaction (Figure 1). Residue K430 forms a pi-cation interaction directly to the benzene ring. Finally, the S3 site is occupied by a (E)‐[(3,4‐dimethoxyphenyl) methylidene]hydrazine moiety. This sub-structure is located near the periphery of the BTK binding site and a hydrophobic interaction occurs between residue L408 and its aromatic ring (Figure 2). The S3 site moiety is also in a position near residue C481, which has been exploited in the past to create covalent BTK inhibitors^37^. However, no interaction with this residue was observed for compound NSC726558. Overall, these interactions suggest that NSC726558 binds to the BTK binding site.

### Evaluation of the anti-cancer activity of NSC726558 in cancer cells

**Figure 3.**
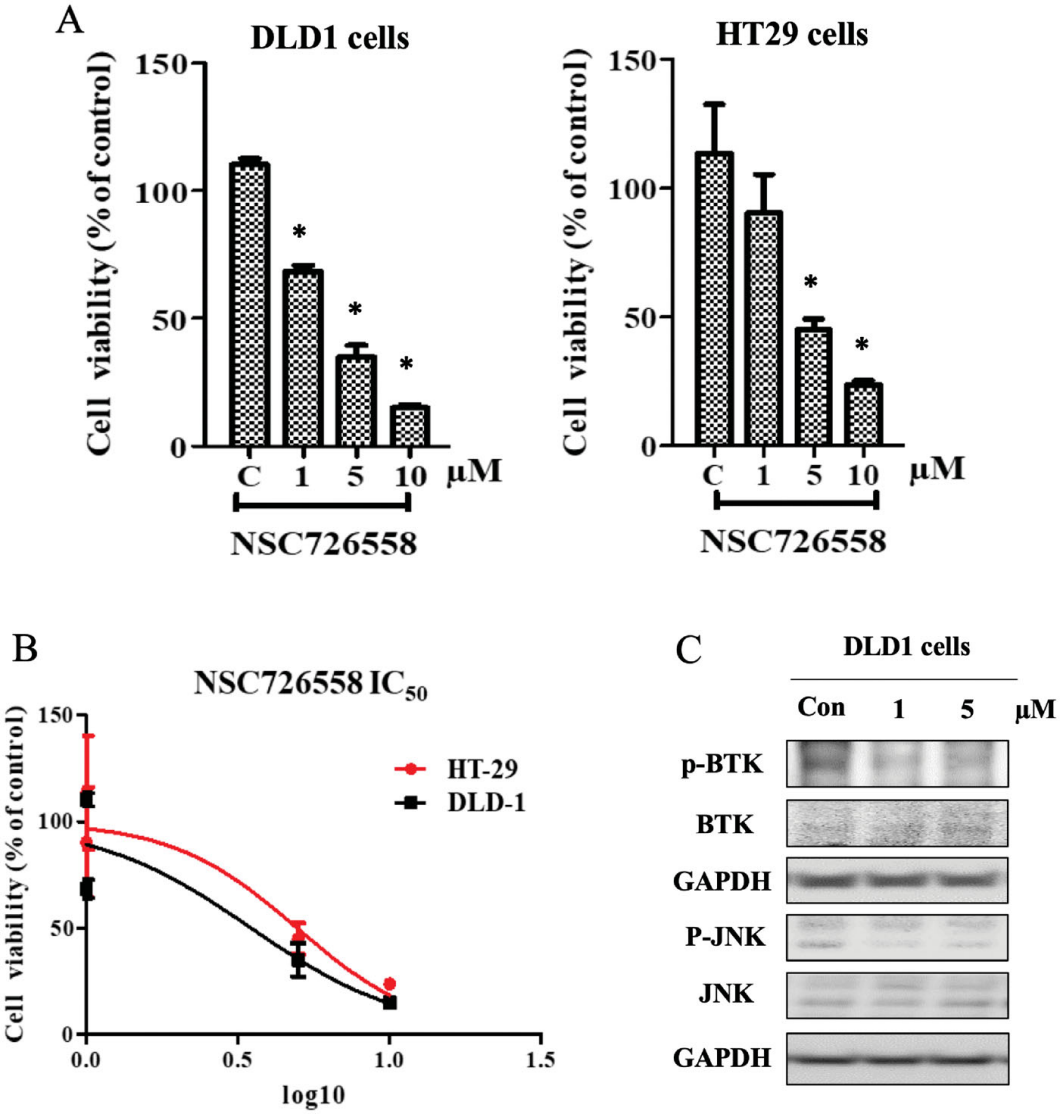
BTK inhibition with compound NSC726558 in cancer cells. (A) Colon cancer cells treated with different concentrations of the compound for 72 h were examined by MTT assay. (B) The IC_50_ of compound NSC726558 in the indicated cancer cells. (C) The western blot assay of cancer cells treated with NSC726558 for 48 h.

There have been reports of elevated BTK expression in solid cancers, such as colorectal cancer^9^^,^^38^. To that end, NSC726558 was evaluated for anti-cancer activity in colorectal cancer cells, DLD1 and HT29. Cancer cells were treated with the compound for 48 h, and then cell viability was examined (Figure 3(A)). Cancer cells treated with compound NSC726558 at concentration of 10 µM showed a significant decrease of cell viability. This effect was observed dose-dependently. The IC_50_ value of NSC726558 was determined as 3.49 and 4.95 µM in DLD1 and HT29 cells, respectively (Figure 3(B)). Cells treated with NSC726558 also show a disruption of BTK expression as well as reducing the phosphorylation of its downstream target, c-Jun N-terminal Kinase (JNK) (Figure 3(C)). To further confirm the cancer cell inhibition capacity of the compound, a calcein-AM staining assay was performed. The calcein-AM is a cell-permeant dye converted by cytosolic esterases in metabolically active cells to produce green fluorescent calcein^39^. In a dose-dependent fashion, NSC726558 showed suppression of cancer cell survival in DLD1 colorectal cells (Supplementary Figure 3). High expression of BTK has been found to be a marker for the development of glioma^6^. The growth of glioblastoma cells U118MG was also suppressed with NSC726558 treatment (Supplementary Figure 3). The above results suggest that NSC726558 functions as a BTK inhibitor and can suppress cancer activity.

### Examination of NSC726558 and its analogues

**Figure 4.**
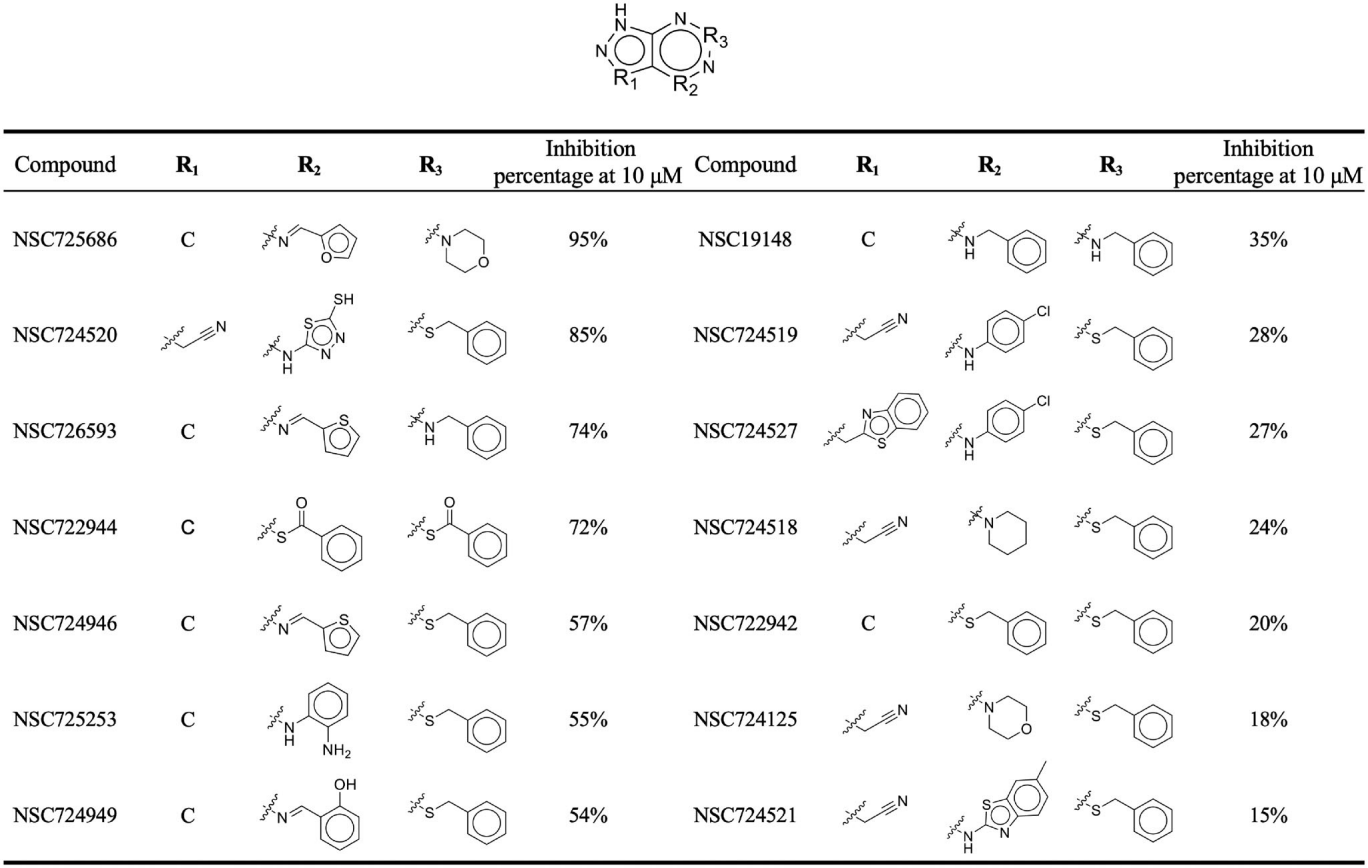
Inhibition assays of NSC726558 analogues. The 14 analogues were tested for BTK inhibitory activity at 10 µM. Active inhibitors produced an inhibition activity ≤ 50%.

Analogues to NSC726558 were obtained from the NCI compound database in order to further elucidate interactions that may induce BTK inhibition. The analogues share a 1H‐pyrazolo[3,4‐d]pyrimidine core (Figure 4). Enzymatic inhibition assay revealed seven analogues with BTK inhibition rate of ≥50%. With an inhibition rate of 95% at 10 µM, NSC725686 was found to be the most potent analogue (Figure 4). Indeed, it displayed an even greater BTK inhibition rate when compared to the inhibition rate of compound NSC726558 (59% at 10 µM) identified previously. The IC_50_ value for compounds exhibiting ≥ 70% inhibitory activity towards BTK was determined by sequential doses (Table 1 and Supplementary Figure 4). Compound NSC725686 displayed the most favourable IC_50_ value of 0.84 µM. Therefore, the analogue NSC725686 has greater potential for BTK inhibitory activity.

**Table 1.** The IC_50_ values of the top four analogues.

Compound	IC_50_ (μM)
NSC725686	0.84
NSC722944	1.60
NSC724520	2.10
NSC726593	5.00

**Figure 5.**
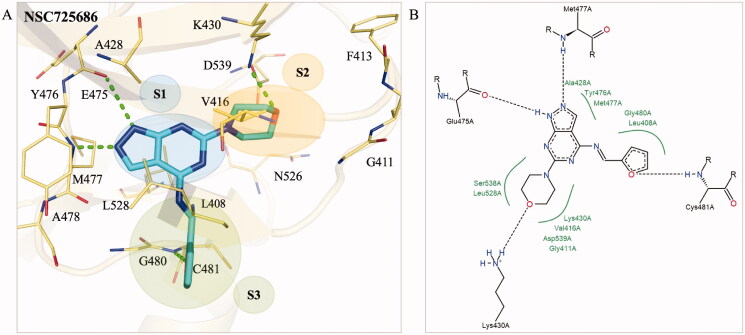
Interaction analysis of analogue NSC725686. (A) The docking pose of analogue NSC725686 (blue) in the BTK binding site (yellow). Green dashes designate hydrogen bonds and residues are labelled and shown as lines. (B) A 2D interaction pose of the analogue NSC725686 was generated. Hydrogen bonds are represented as black dashes and hydrophobic pockets as green splines.

To elucidate the binding mechanism of compound NSC725686, we performed an interaction analysis of its docking pose. Both compounds share a 1H‐pyrazolo[3,4‐d]pyrimidine core that occupies the S1 site to form hydrogen bonds with residues E475 and M477 (Figure 5). This core also occupies a hydrophobic pocket at the S1 site that includes residue A428, Y476, and M477 (Figure 5(B)). The S2 site is occupied by a morpholine moiety that forms a hydrogen bond with residue K430 (Figure 5). Compared to the hit compound NSC726558, the morpholine moiety of compound NSC725686 does not extend into hydrophobic pocket of S2. This may be due to the longer linker displayed on compound NSC726558. Nevertheless, the morpholine structure makes hydrophobic interactions with residues K430 and L528 and a hydrogen bond to K430 (Figure 5(B)). The S3 pocket is occupied by the (furan‐2‐yl)methanimine moiety that lies at the periphery of the BTK binding site. The S3 sub-structure spans a sub-pocket that is made up of residues L408 and G480. Key hydrophobic interactions are formed between the S3 sub-structure and residue L408 and C481 (Figure 5(B)). A hydrogen bond is formed between the oxygen on the furan ring and residue C481 (Figure 5(B)). The four key hydrophobic interactions identified earlier were present between NSC725686 and the BTK binding site, suggesting sufficient binding interactions to residues.

Next, a comparison of the interactions between the active compounds identified in this study and the inactive analogues was performed. Seven analogues produced a BTK inhibition ≥ 50% and, along with the selected inhibitor NSC726558, were labelled as active compounds (Supplementary Figure 5(A)). All active compounds form hydrogen bonds to hinge residues E475 and M477. Several active compounds show a hydrogen bond to residue C481. Current covalent BTK inhibitors form an irreversible bond to residue C481. However, this covalent bond is non-specific and can bind to other cysteine-containing kinases, which may reduce its effectiveness for treating solid tumours^7^^,^^13^^,^^14^. A hydrogen bond to the cysteine may prove effective for BTK inhibition. Indeed, the most potent analogue, NSC725686, was observed to have this hydrogen bond interaction. For example, the active compound, NSC726593, occupies the S3 site with a thiophene moiety, a five-membered ring (Supplementary Figure 5(E)). This moiety forms both hydrophobic and hydrogen-bonding interactions with residue C481 (Supplementary Figure 5(A)). Analogue NSC726593 showed a BTK inhibition percentage of 74% (Figure 4). Comparing the active analogue NSC726593 with the inactive analogues, the five-membered ring moiety may account for greater BTK inhibition. The active analogue NSC725686 displayed the best BTK inhibition at 95% (Figure 4). This may be due to its additional hydrophobic alkyl interaction with residues V416 and K430. This type of interaction is not present with any of the other analogues, nor with the identified BTK inhibitor NSC726558 (Supplementary Figure 5(A)).

Analysis of the inactive inhibitors revealed a dearth of interactions in other areas when compared to the active compounds. Interestingly, the inactive compounds also form at least one hydrogen bond to either hinge residues. These interactions are generated due to the shared scaffold of the analogues. The hydrogen bond to residue C481 was not observed with the inactive analogues. Many of the inactive compounds contain larger ring moieties that may prevent them from occupying or forming interactions at the S3 site. For example, compound NSC724125 contains a morpholine moiety that occupies the S3 site, but is not located at a sufficient distance for interactions with residue C481 (Supplementary Figure 5(B)). The inactive compound NSC19148 contains a benzene ring that extends into site S3 and forms hydrophobic interaction with residue C481, but no hydrogen bond is formed (Supplementary Figure 5(C)). The weakest analogue, NSC724521, contains a large benzothiazole ring that does not extend into site S3 (Supplementary Figure 5(D)). This suggests that, for these scaffolds, a smaller ring structure with a polar atom is needed to form either a hydrophobic interaction or hydrogen bond to residue C481 for greater BTK potency. Thus, these moieties on the inactive compounds are not conducive for interactions at the S3 site.

Approved BTK inhibitors have been developed for oral administration^5^. To determine oral bioavailability of the top four analogous with favourable BTK inhibitory activity, predictions regarding their absorption, distribution, metabolism, excretion, and toxicity was performed (Supplementary Table 2). The compounds fit the Lipinski rule of five, suggesting that these compounds have favourable properties for oral bioavailability. Absorption of an oral drug occurs with the gastrointestinal system. The human intestinal absorption for a particular drug is an important property to gauge whether a small molecule can be delivered to its target^40^. Three of the compounds show favourable absorption levels (Supplementary Table 2). Further, the most potent compound, NSC725686, has the most favourable QED score, suggesting that it is the most drug-like compound. Compound NSC725686 contains an imine linker that may be cleaved in an acidic environment of the gastrointestinal tract. Exploring different formulations may be a strategy to avoid hydrolysis and to maintain potency. Our docking analysis suggests that the imine group does not make significant interactions to the target binding site (Figure 5). Further studies would explore modifications to the imine linker and optimisation of NSC725686 as a potential BTK inhibitor.

### Investigation of the suppression capacity and molecular mechanisms of NSC725686 in cancer cells

Further testing was performed to elucidate the cancer suppression capacity of the active compounds. Ibrutinib, the first-generation FDA-approved BTK inhibitor, has been found to exhibit anti-tumour activity in not only B-cell-related cancers but also in other types of cancers, such as glioblastoma and skin cancer, through apoptosis, and autophagy pathways^41^^,^^42^. Apoptosis induction mediated by BTK suppression has been shown in colon cancer previously^8^^,^^43^. However, whether BTK inhibitors can exhibit anti-cancer capacity through autophagy pathway in colon cancer was unclear and yet to be illustrated.

**Figure 6.**
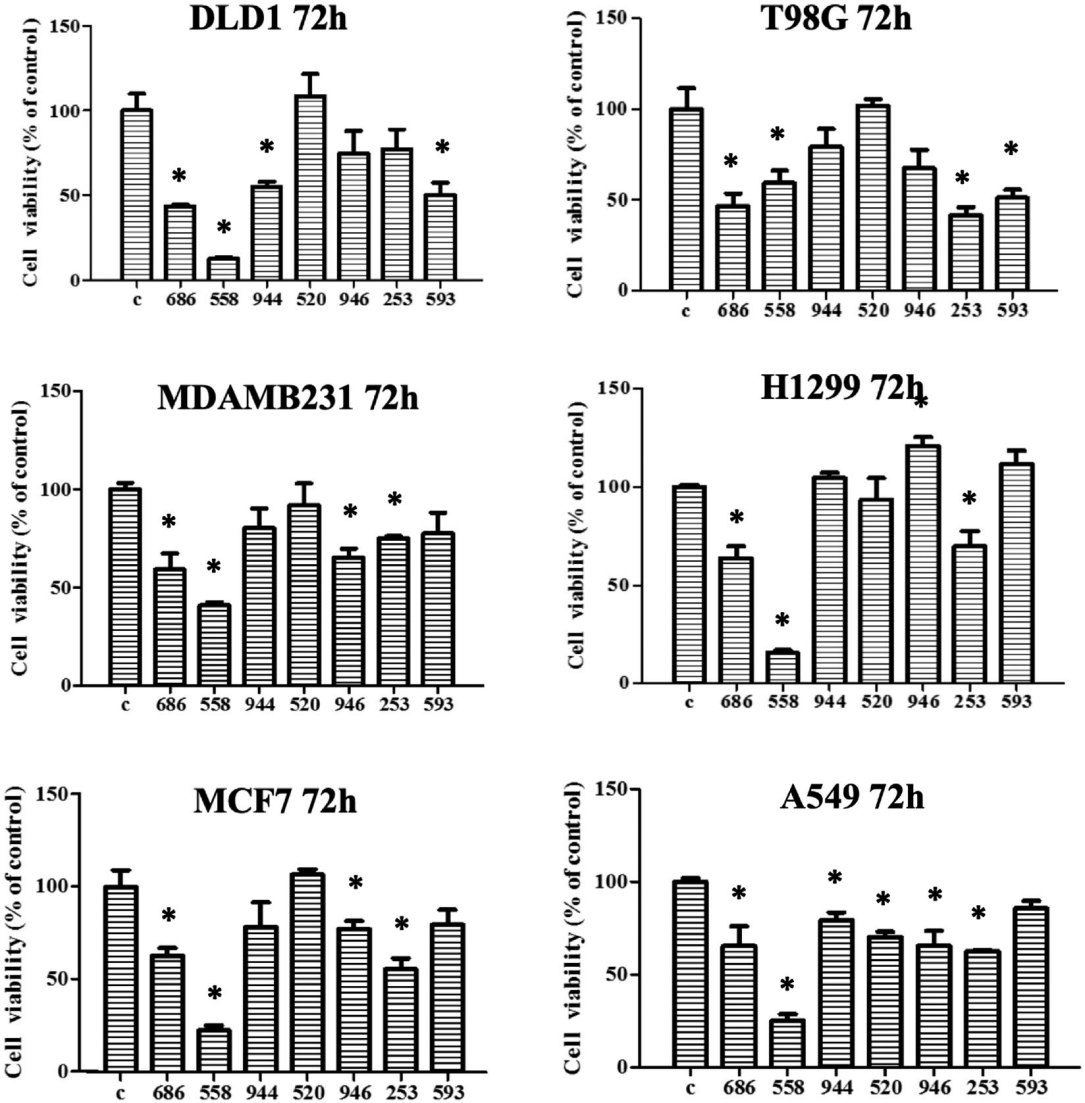
Compound NSC726558 shows potency in different cancer cell lines. A MTT assays of NSC726558 analogues was performed. The cancer cell lines were treated with 20 µM of the indicated compounds for 72 h and then analysed for cell viability. Colon cancer cells (DLD1), glioblastoma cells (T98G), breast cancer cells (MDAMB231 and MCF7), and lung cancer cells (H1299 and A549) were applied in the experiment.

Cell lines representing various cancers, such as glioblastoma, lung, breast, and colon, were treated with the indicated compounds for 72 h (Figure 6). The numbers of the compounds were shortened as the last three-digit numbers as indicated in the figures. Of the compounds tested, the identified BTK inhibitor NSC726558 exhibited the strongest cancer inhibition (Figure 6). Its analogue, NSC725686, also showed favourable cancer inhibition. Moreover, as in Supplementary Figure 3, glioblastoma cells could be suppressed with NSC726558 treatment. Treatment with NSC726558 or NSC725686 also displayed cancer inhibition capacity towards glioblastoma cells T98G (Figure 6). The above results suggest that our BTK inhibitors could suppress cancer activity of solid tumours.

To determine toxicity of these compounds towards normal cells, the compounds were evaluated in the non-cancerous kidney cell line 293 T. NSC726558 exhibited severe toxicity in 293 T cells compared to the other compounds, which suggests that it may not be a suitable lead compound (Supplementary Figure 6). In contrast, the analogue NSC725686 shows less toxicity in the assay. This suggests that compound NSC725686 may serve as a better candidate for further optimisation.

The compound NSC725686 was selected for further assays to determine its cytotoxicity towards cancer cells. Live and dead assay was performed using two dyes, calcein-AM for staining live cells and PI for staining dead cells. Cells were treated with compound NSC725686 for 48 h. The results displayed a significant PI signal, while the reduction of calcein-AM was less obvious (Supplementary Figure 7). This suggests that a cell death pathway may be induced with NSC725686 treatment.

To further confirm the induction of cell death, we analysed the apoptosis and autophagy pathways of cancer cells under the NSC725686 treatment. Cancer cells were treated with NSC725686 for 48 h, and then harvested for western blot analysis. The expression of apoptosis markers, PAPR, and Caspase 3 cleavage, was increased when treated with NSC725686 (Supplementary Figure 8(A)). Moreover, cancer cells treated with NSC725686 had increased population of sub-G1 cells, suggesting an induction of apoptosis (Supplementary Figure 8(B)). Finally, to examine possible induction of autophagy, cancer cells were treated with the indicated compounds for 48 h and then harvested for western blot analysis. The autophagy marker LC3B in cancer cells treated with compounds NSC726558 or NSC725686 displayed increased expression (Supplementary Figure 9). Accumulation of LC3B puncta signal in cancer cells was observed with NSC725686 treatment, indicating the formation of autophagosomes and promotion of the autophagy pathway (Supplementary Figure 9(B)). These results demonstrate that NSC725686 suppressed cancer cells through induction of apoptosis and autophagy pathways.

### NSC725686 is a novel BTK inhibitor

Next, we compared the structure of the identified inhibitor, NSC725686, to the known BTK inhibitors obtained from BindingDB. A novel inhibitor could function as a starting point for designing or optimising for greater BTK selectivity. The structural analysis contained a total of 30 known BTK inhibitors with diverse scaffolds. A hierarchal clustering and Pearson’s correlation analysis were used to measure the similarity between the compound structures and plotted as a heatmap (Supplementary Figure 10(A)). Compound NSC725686 showed little similarity with the known BTK inhibitors. The most similar compound contained a score no greater than 0.42 (Supplementary Figure 10(B)). As a novel structure, NSC725686 may serve as a starting point for further optimisation for BTK potency or selectivity.

## Conclusion

The overexpression of BTK has been linked to various solid cancers, making it a viable therapeutic target. Unfortunately, known FDA-approved BTK inhibitors contain a covalent bonding mechanism that may hinder their selectivity and leading to unwanted side effects. In this study, a SBVS approach was used to identify novel small-molecule inhibitor targeting BTK. Analysing the hit compound, NSC726558, and its analogues lead to the identification of NSC725686. Both compounds share a 1H‐pyrazolo[3,4‐d]pyrimidine kinase scaffold. Importantly, NSC725686 showed BTK inhibition (95%) at 10 µM as well as the most IC_50_ value at 0.84 µM. The growth of colorectal, lung, breast, and glioblastoma cell lines was inhibited when treated with NSC725686. The inhibition of the cancer cells may be induced by BTK inhibition, which triggers the apoptosis and autophagy pathways. The results demonstrated the potential of NSC725686 for cancer therapeutics. Altogether, the structure of NSC725686 can serve as a basis for further optimisation for cytotoxicity and potency.

## Supplementary Material

Supplemental Material
