# Supplementary material for: Structure-based virtual screening and biological evaluation of novel small-molecule BTK inhibitors
Source: J Enzyme Inhib Med Chem. 2021 Dec 11;37(1):226–35. doi: 10.1080/14756366.2021.1999237 (PMC8667945; doi:10.1080/14756366.2021.1999237)
Supplement: Supplemental Material [file IENZ_A_1999237_SM9541.pdf]

Supplementary Figures

Structure-based virtual screening and biological evaluation of novel small-molecule BTK inhibitors

Tony Eight Lin[a,b,1], Li-Chin Sung[c,d,1], Min-Wu Chao[e], Min Li[f], Jia-Huei Zheng[f], Tzu-Ying Sung[g], Jui-Hua Hsieh[h], Chia-Ron Yang[e], Hsueh-Yun Lee[f], Er-Chieh Cho,[f,i,j,*], Kai-Cheng Hsu[a,j,k,l,m,n,*]

[a]Graduate Institute of Cancer Biology and Drug Discovery, College of Medical Science and Technology, Taipei Medical University, Taipei, Taiwan
[b]Master Program in Graduate Institute of Cancer Biology and Drug Discovery, College of Medical Science and Technology, Taipei Medical University, Taipei, Taiwan
[c]Division of Cardiology, Department of Internal Medicine, Shuang Ho Hospital, Taipei Medical University, New Taipei City, Taiwan.
[d]Division of Cardiology, Department of Internal Medicine, School of Medicine, College of Medicine, Taipei Medical University, Taipei, Taiwan.
[e]School of Pharmacy, College of Medicine, National Taiwan University, Taipei, Taiwan.
[f]School of Pharmacy, College of Pharmacy, Taipei Medical University, Taipei, Taiwan.
[g]Biomedical Translation Research Center, Academia Sinica, Taipei, Taiwan.
[h]Division of the National Toxicology Program, National Institute of Environmental Health Sciences, National Institutes of Health, Durham, NC, United States.
[i]Master Program in Clinical Genomics and Proteomics, College of Pharmacy, Taipei Medical University, Taipei, Taiwan.
[j]Cancer Center, Wan Fang Hospital, Taipei Medical University, Taiwan.
[k]Ph.D. Program in Drug Discovery and Development Industry, College of Pharmacy, Taipei Medical University, Taipei, Taiwan
[l]TMU Research Center of Cancer Translational Medicine, Taipei Medical University, Taipei, Taiwan
[m]TMU Research Center of Drug Discovery, Taipei Medical University, Taipei, Taiwan
[n]Ph.D. Program for Cancer Molecular Biology and Drug Discovery, College of Medical Science and Technology, Taipei Medical University, Taipei, Taiwan

*Corresponding authors
E-mail: piki@tmu.edu.tw (K.C. Hsu)
E-mail: echo@tmu.edu.tw (E.C. Cho)

[1]These authors contributed equally to this work.

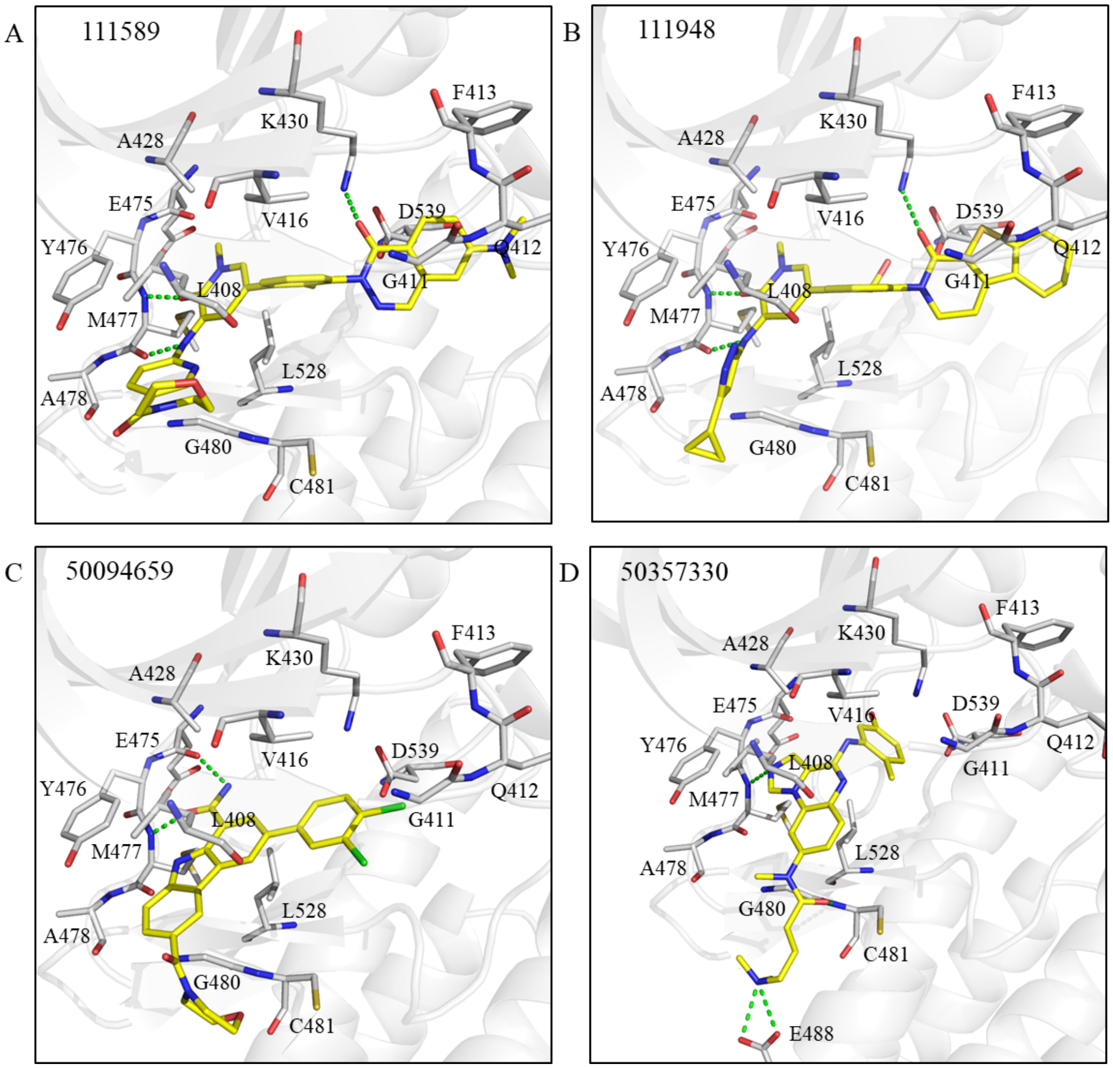

Supplementary Figure 1. Known inhibitors docked into the BTK binding site. The docking poses of compounds (A) 111589, (B) 111948, (C) 50094659 and (D) 50357330 in the BTK binding site. Hydrogen bonds are denoted as a dashed green line. Inhibitors are colored yellow. BTK is colored gray. Residues are listed as shown.

Compounds	Inhibition percentage at 10 μM	Compounds	Inhibition percentage at 10 μM	Compounds	Inhibition percentage at 10 μM
NSC726558 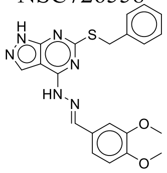	59	NSC658345 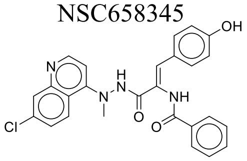	13	NSC659393 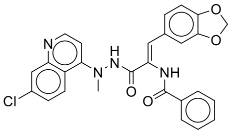	8
NSC666713 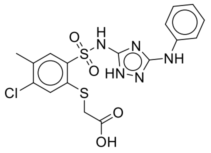	30	NSC151998 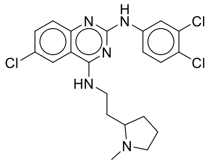	11	NSC82640 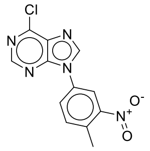	7
NSC526958 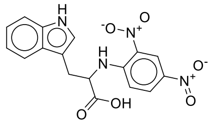	30	NSC63665 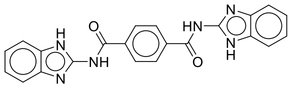	11	NSC368390 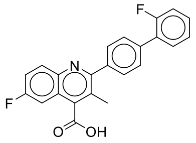	6
NSC124210 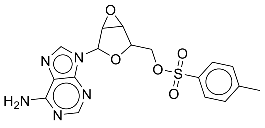	24	NSC346587 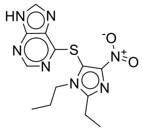	8		

Supplementary Figure 2. Inhibition assays of potential inhibitors. The identified compounds were tested for BTK inhibition at 10 μM.

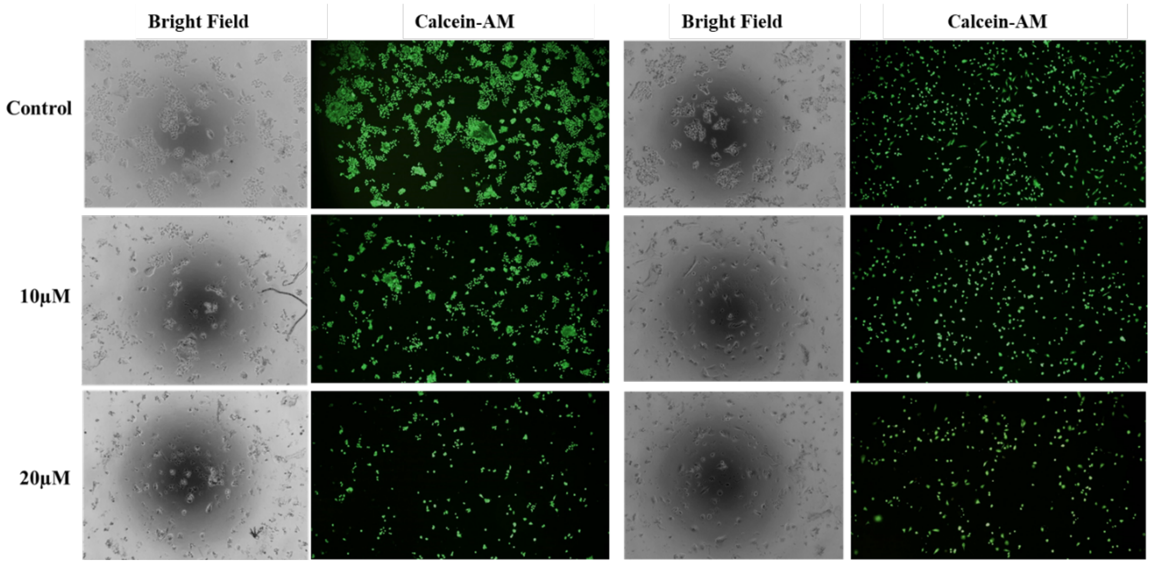

Supplementary Figure 3. Calcein-AM staining for DLD1 & U118MG cells under NSC726558 treatment. Cells (DLD1 in the left panel and U118MG in the right panel) were treated with the compound for 48h, stained by calcein-AM, and analyzed under the fluorescence microscopy.

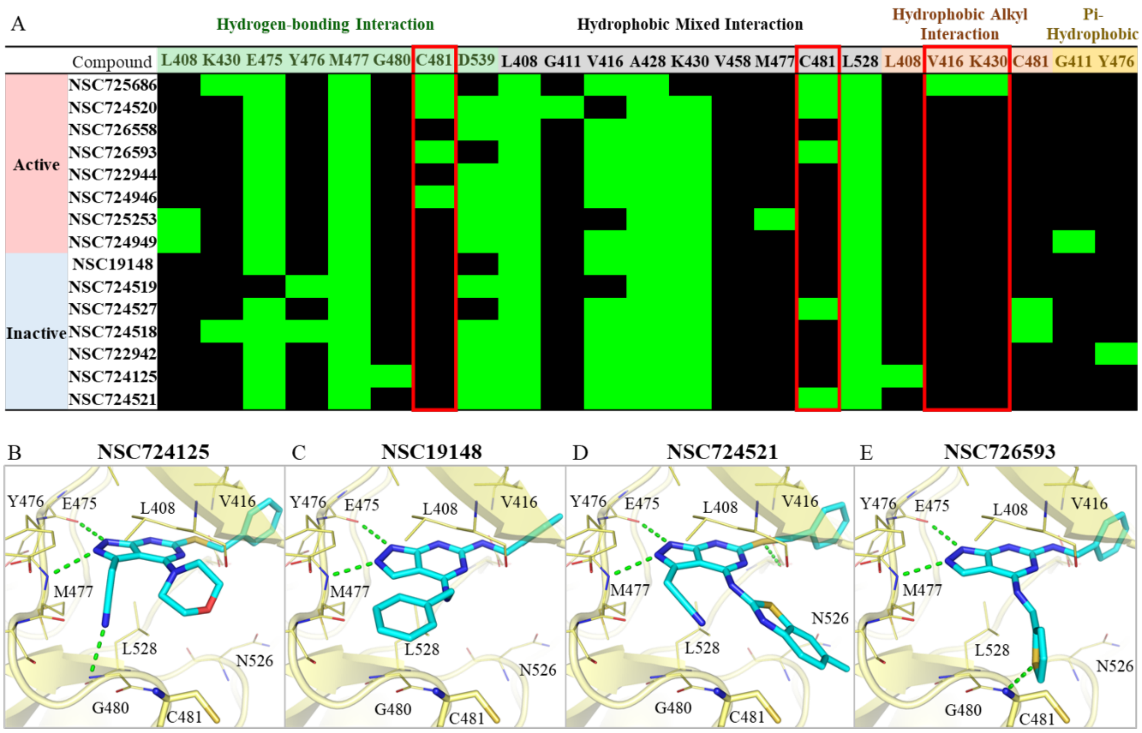

Supplementary Figure 4. Interaction analysis of analogs. (A) The heatmap indicates interactions between the compounds and BTK residues. Green denotes interaction with indicated residue. The docking poses of the inactive compounds (B) NSC724215 (C) NSC19148 and (D) NSC724521 and the active compound (E) NSC726593 in BTK are shown. Green dashes indicate hydrogen bonds. Residues are listed and represented as lines.

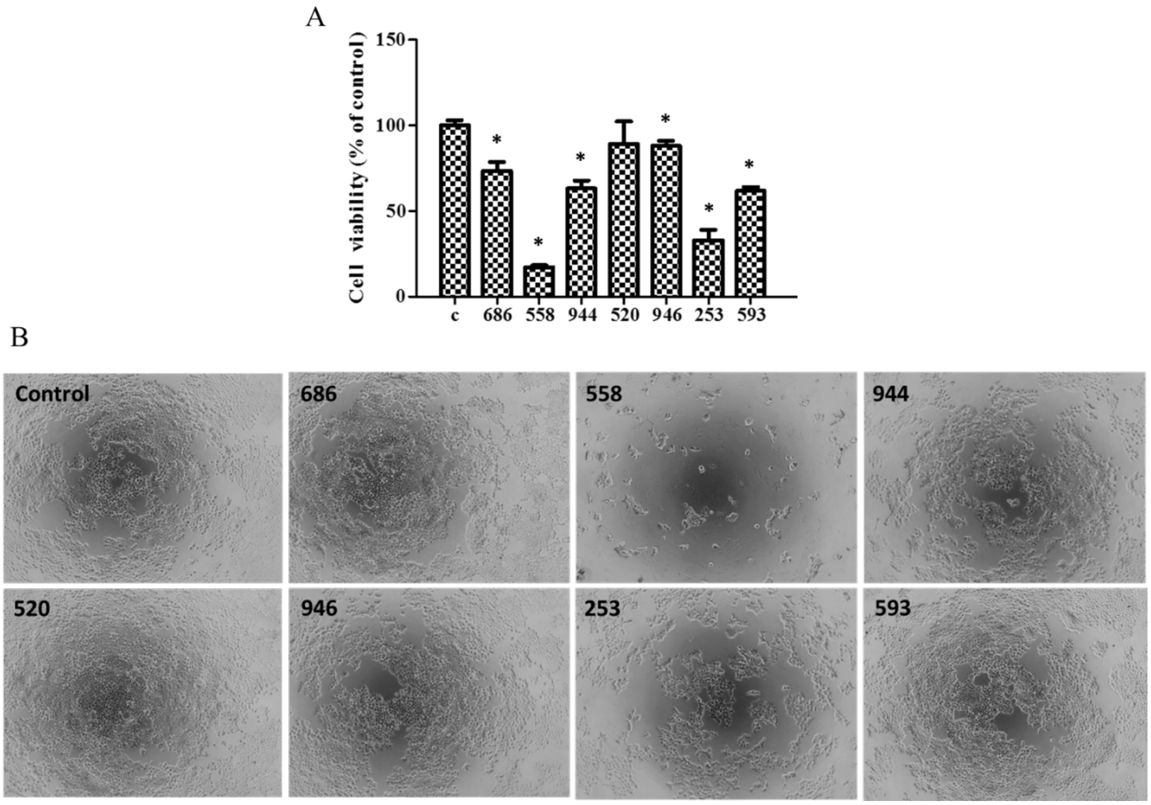

Supplementary Figure 5. Evaluation of NSC726558 analogs in non-cancerous 293T cells. (A) Selected compounds were treated at 20 μM concentration for 48h, and then analyzed by MTT assay to evaluate the cell viability and compound safety. (B) Observation for cell morphology was performed under microscopy.

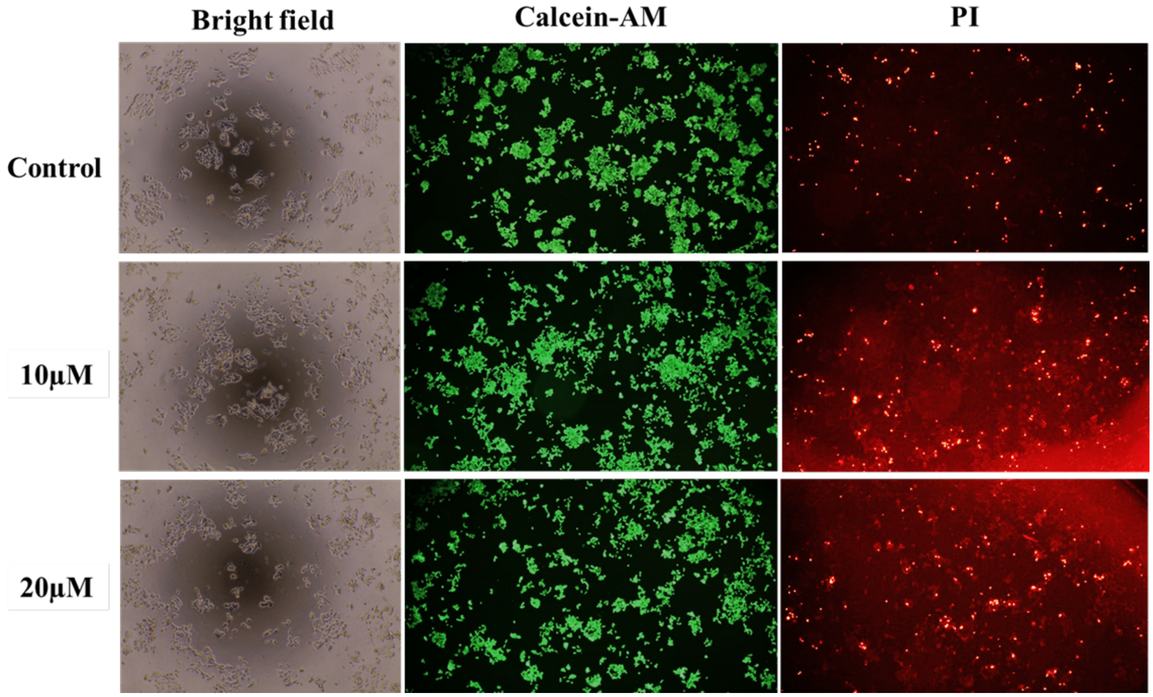

Supplementary Figure 6. The live and dead assay of HCT116 under NSC725686 treatment. Cells were treated with the compound for 48h, stained with calcein-AM and PI as described in the materials and methods, and then analyzed under the fluorescence microscopy.

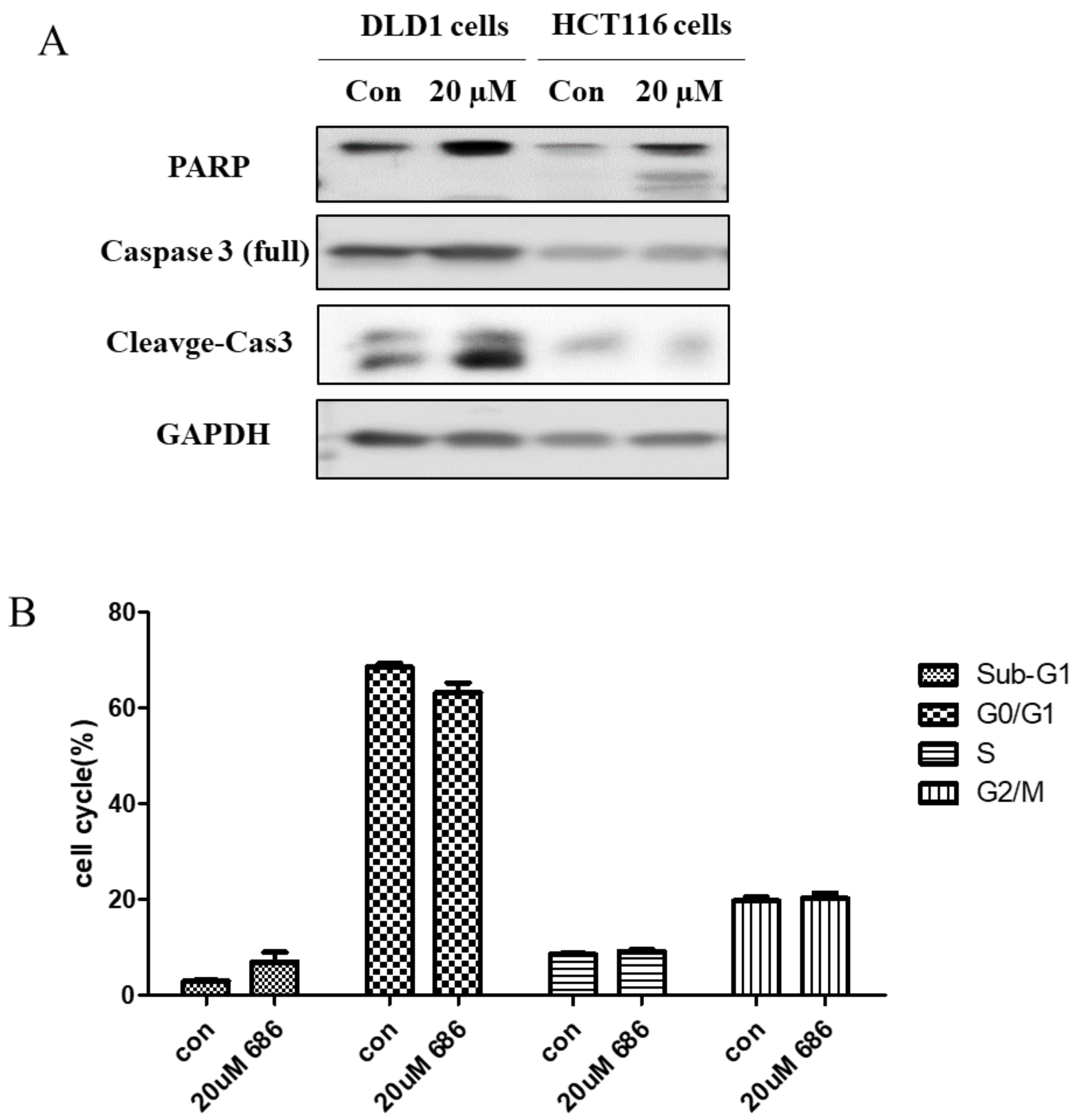

Supplementary Figure 7. NSC725686 induced apoptosis pathway in cancer cells. (A) Cells were treated with the compound at 20 µM concentration for 48h, harvested, and analyzed by western blot assay with indicated antibodies. (B) Flow cytometry assay was performed to identify cell cycle arrest.

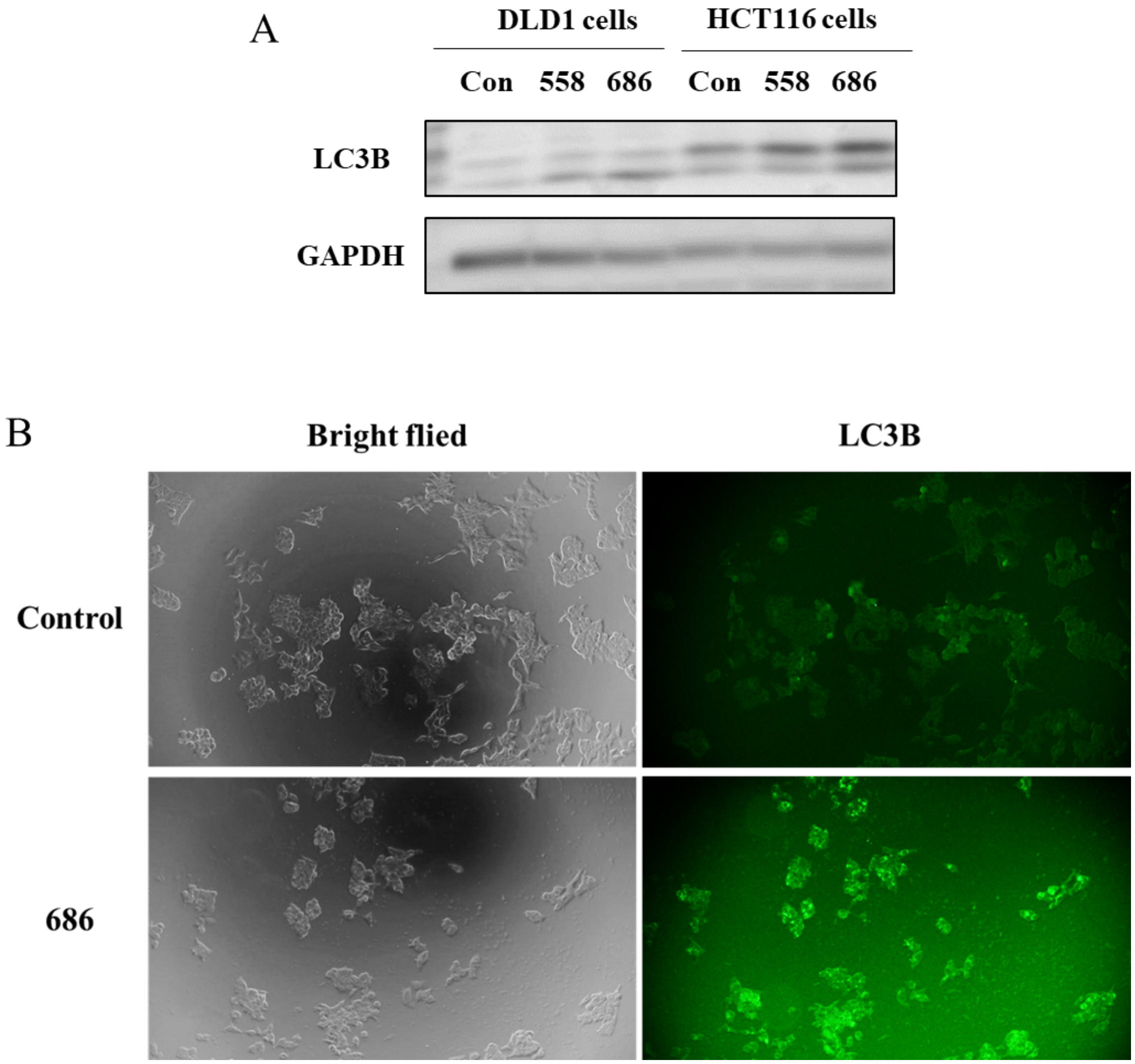

Supplementary Figure 8. NSC726558 and NSC725686 induced autophagy pathway in cancer cells. (A) Cells were treated with the NSC726558 (10 µM) and NSC725686 (20µM) for 48h, harvested, and analyzed by western blot assay with LC3B antibody. (B) HCT116 cells were treated with NSC725686 (20 µM) for 48h and analyzed by immunofluorescence assay with LC3B antibody.

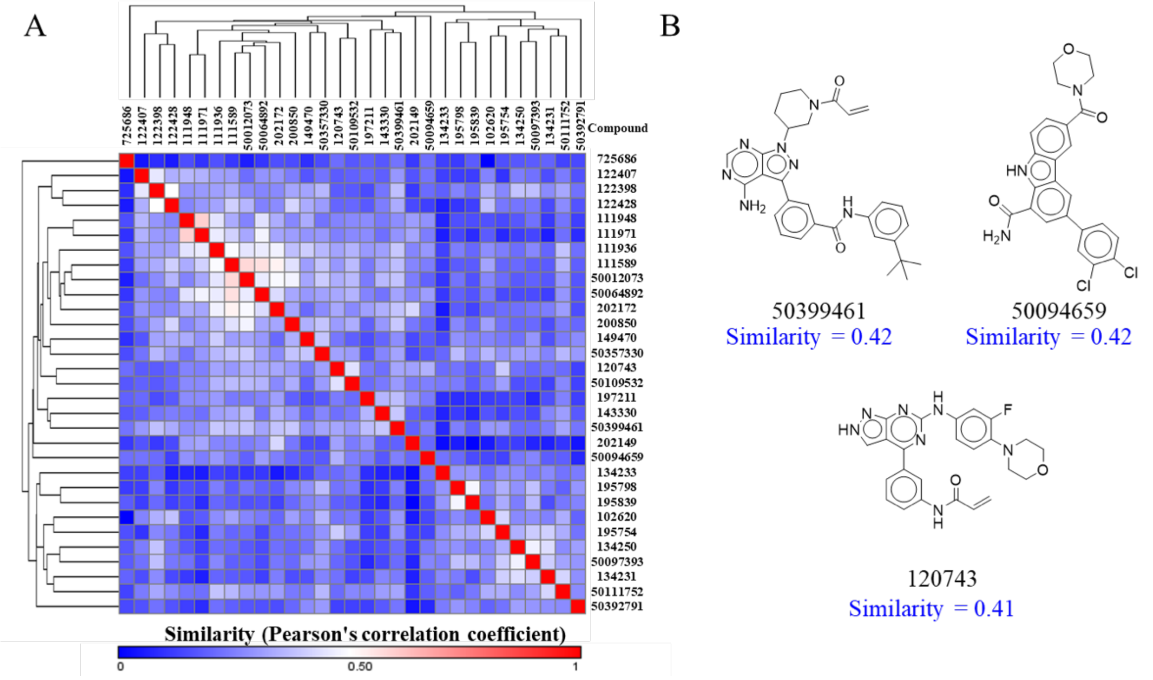

Supplementary Figure 9. Structure comparison of NSC725686 and other BTK inhibitors. (A) A heatmap presenting similarities between compounds. The structure of the active inhibitor was compared against 30 diverse BTK inhibitors. A score of 1 (red) to 0 indicates similarity or dissimilarity, respectively. (B) The structures of the most similar compounds compared to NSC725686. A similarity score for each structure is listed.

Supplementary Table 1. The enrichment factor value of the virtual screening protocol.

Percentage of active compounds	Enrichment factor
10%	11.33
20%	2.96
30%	3.78
40%	4.39
50%	4.86
60%	5.1
70%	5.41
80%	4.61
90%	4.43
100%	2.31

Supplementary Table 2. The molecular properties of the top four analogous.

Compound	Absorption level[a]	Molecular weight	ALogP	Number of rotatable bonds	Number of hydrogen bond acceptors	Number of hydrogen bond donors	RO5[b] Status	QED[c] score
NSC725686	0	298.3	1.8	3	6	1	PASS	0.74
NSC722944	0	392.5	4.9	6	7	1	PASS	0.28
NSC724520	2	412.5	4.3	6	9	3	PASS	0.23
NSC726593	0	334.4	3.9	5	5	2	PASS	0.54

[a] Absorption levels range from 0 (Good) to 3 (Very Low).
[b] RO5: Rule of Five.
[c] QED: Quantitative Estimate of Drug-likeness.
